# A Bivalent Human Adenovirus Type 5 Vaccine Expressing the Rabies Virus Glycoprotein and Canine Distemper Virus Hemagglutinin Protein Confers Protective Immunity in Mice and Foxes

**DOI:** 10.3389/fmicb.2020.01070

**Published:** 2020-06-16

**Authors:** Lina Yan, Zhongxin Zhao, Xianghong Xue, Wenwen Zheng, Tong Xu, Lele Liu, Li Tian, Xianwei Wang, Hongbin He, Xuexing Zheng

**Affiliations:** ^1^Department of Virology, School of Public Health, Cheeloo College of Medicine, Shandong University, Jinan, China; ^2^Divisions of Infectious Diseases of Special Animal, Institute of Special Animal and Plant Sciences, Chinese Academy of Agricultural Sciences, Changchun, China; ^3^School of Life Sciences, Shandong University, Qingdao, China; ^4^College of Life Sciences, Shandong Normal University, Jinan, China

**Keywords:** human adenovirus 5, rabies virus, canine distemper virus, glycoprotein, hemagglutinin, bivalent vaccine

## Abstract

The development of a safe and efficient multivalent vaccine has great prospects for application. Both rabies virus (RABV) and canine distemper virus (CDV) are highly infectious antigens, causing lethal diseases in domestic dogs and other carnivores worldwide. In this study, a replication-deficient human adenovirus 5 (Ad5)-vectored vaccine, rAd5-G-H, expressing RABV glycoprotein (G) and CDV hemagglutinin (H) protein was constructed. The RABV G and CDV H protein of rAd5-G-H were expressed and confirmed in infected HEK-293 cells by indirect immunofluorescence assay. The rAd5-G-H retained a homogeneous icosahedral morphology similar to rAd5-GFP under an electron microscope. A single dose of 10^8^ GFU of rAd5-G-H administered to mice by intramuscular injection elicited rapid and robust neutralizing antibodies against RABV and CDV. Flow cytometry assays indicated that the dendritic cells and B cells in inguinal lymph nodes were significantly recruited in rAd5-G-H-immunized mice in comparison with the mock and rAd5-GFP groups. rAd5-G-H also activated the Th1- and Th2-mediated cell immune responses against RABV and CDV in mice, which contributed to 100% survival of a lethal-dose RABV challenge without any clinical signs. In foxes, a single dose of 10^9^ GFU of rAd5-G-H could elicit high levels of neutralizing antibodies against both RABV and CDV in comparison with the mock and rAd5-GFP groups. All foxes in the rAd5-GFP and mock groups died, while the foxes inoculated with rAd5-G-H all survived and showed no clinical signs of disease after being challenged with a lethal wild-type CDV strain. These results suggested that rAd5-G-H has great potential as a bivalent vaccine against rabies and canine distemper in highly susceptible dogs and wildlife animals.

## Highlights

– A recombinant replication-defective human adenovirus simultaneously expressing the RABV G protein and CDV H protein, rAd5-G-H, was generated.
– rAd5-G-H, significantly recruiting and activating DCs and persistent B cells, elicited both robust humoral and cell-mediated immune responses against RABV and CDV in mice with 100% survival of a fatal RABV challenge in mice.
– rAd5-G-H showed robust humoral immune responses against RABV and CDV in foxes and conferred complete protection against a lethal wild-type CDV strain challenge in foxes.

## Introduction

Both rabies virus (RABV) and canine distemper virus (CDV) cause fatal diseases in carnivores. Rabies poses a great threat to global public health. It is estimated that about 59,000 humans from 150 countries die of rabies each year (World Health Organization, 2019). The majority (95%) of cases are transmitted from the bites or scratches of infected animals and occur in developing countries in Africa and Asia, especially in rural areas, where animal rabies vaccinations are not paid enough attention (Jackson, 2013; World Health Organization, 2013). Almost all mammals are susceptible to rabies, and a comparatively small number of carnivores, mainly dogs, foxes, raccoon dogs, raccoons, skunks, and mongooses, serve as wildlife reservoirs (Wirblich and Schnell, 2011). Vaccinations of the primary terrestrial species are the most effective method of controlling rabies. The glycoprotein (G) of a pathogenic RABV strain determines viral neuro-tropism and retrograde trans-synaptic spread and mediates fatal encephalitis (Scott and Nel, 2016). However, the G protein of a non-pathogenic RABV, as a major antigen determinant, plays a critical role in eliciting virus-neutralizing antibodies (VNAs) and developing protective host immune responses (Jackson, 2013; Astray et al., 2017). virus-neutralizing antibody levels higher than 0.5 IU/mL are considered to be sufficient to protect susceptible species from rabies infection, since the VNAs are mainly directed against the surface G protein of the RABV (Zhao et al., 2019). Therefore, the design of a novel rabies vaccine focuses on the RABV G protein (Astray et al., 2017).

Canine distemper is a highly contagious disease caused by its agent, CDV, a member of the *Morbillivirus* genus within the family *Paramyxoviridae* (Deem et al., 2000; Li et al., 2018). Canine distemper virus infects a broad range of host carnivores worldwide, even non-human primates (Feng et al., 2016; Martinez-Gutierrez and Ruiz-Saenz, 2016), causing severe immunosuppression and multi-systemic clinical signs including in the respiratory, gastrointestinal, and central nervous systems (von Messling et al., 2004; Xue et al., 2019). Although CDV vaccines have been routinely administered to dogs for many years, canine distemper remained a major contagious disease in domestic dogs as well as in wildlife animals (Romanutti et al., 2016). The hemagglutinin (H) protein of CDV, a critical antigen determinant, mediates attachment of virions and their entry into host cells by binding to the signaling lymphocyte activation molecule (SLAM) in lymphocytes (Tatsuo et al., 2001) or nectin-4 receptor in epithelial cells (Noyce et al., 2013). Many studies have proved that the H protein plays a pivotal role in eliciting the protective immune response during CDV infection (Bhatt et al., 2019). The H protein contains cytotoxic T lymphocyte epitopes that induce cytotoxic T lymphocytes (CTL) antiviral activity in animals and has become a target for developing novel genetically engineered vaccines (Rendon-Marin et al., 2019).

The inactivated rabies vaccines have been applied in human and dogs for many years; however, they are expensive and need multiple inoculations to elicit effective humoral immune responses (El-Sayed, 2018). Live attenuated rabies vaccines raise safety concerns about virulence reversion and are no longer available commercially in China (Zhang W. et al., 2019). In addition, live-attenuated CDV vaccines might retain residual virulence in highly susceptible species (Rendon-Marin et al., 2019). Therefore, a safe and efficient bivalent vaccine against rabies and canine distemper needs to be generated. The replication-defective adenovirus type 5 (rAd5) vector has been widely used for vaccine research, having the advantages of being safe and efficacious and having a high-capacity to simultaneously express various exogenous genes (Tatsis and Ertl, 2004). Replication-defective adenovirus type 5 has been extensively explored as a vaccine vector for a variety of pathogens, such as HIV (Churchyard et al., 2011), influenza virus (Gao et al., 2006), RABV (Vos et al., 2001), *Mycobacterium tuberculosis* (Smaill et al., 2013), dengue virus (Khanam et al., 2009), and Middle East respiratory syndrome coronavirus (Guo et al., 2015). In addition, a rAd5-based human vaccine expressing Ebola G protein was approved for new drug registration by the Chinese Food and Drug Administration (FDA) in 2017 (NCT02326194) (Zhu et al., 2017). Therefore, in the present study, the recombinant replication-defective virus rAd5-G-H, with co-expression of the RABV G protein and CDV H protein, is generated to serve as a bivalent vaccine against RABV and CDV.

## Materials and Methods

### Cells, Viruses, Antibodies, and Animals

HEK-293, BHK-21, mouse neuroblastoma (NA), and Vero cells were all cultured at 37°C in Dulbecco’s modified Eagle’s medium (DMEM; Gibco, Invitrogen, China) supplemented with 10% fetal bovine serum (Gibco, Grand Island, NY, United States). The vaccine RABV (SRV9 strain), standard challenge RABV (CVS-11 strain), and street RABV (HuNPB3 strain) were all propagated in NA cells. The vaccine CDV (CDV-11 strain) was propagated in Vero cells. The rAd5-GFP and rAd5-G-H were propagated in HEK-293 cells. Mouse anti-RABV G monoclonal antibody was purchased from Millipore (MA, United States). Goat anti-CDV polyclonal antibody was purchased from VMRD (Washington, DC, United States). Donkey anti-mouse IgG and donkey anti-goat IgG secondary antibodies (Alexa Fluor^®^ 488) were purchased from Abcam (Cambridge, United Kingdom). Antibodies used for flow cytometry assays, such as PE anti-mouse CD86, FITC anti-mouse MHC I, PE anti-mouse MHC II, PE Cy^TM^7 anti-mouse CD11c, APC anti-mouse CD19, and FITC anti-mouse CD40, were purchased from BD Biosciences (Franklin, TN, United States).

Female BALB/c mice (6–8 weeks old, weighing 17–19 g) were obtained from Pengyue Animal Breeding Center (Jinan, China). Female foxes (8 weeks old) were purchased from Qilu Pharmaceutical Co. Ltd. (Jinan, China). All of the mice and foxes were serologically negative for both RABV and CDV. The animal treatments and sample preparations complied with the Animal Ethics Procedures and Guidelines and were approved by the Institutional Animal Care and Use Committee (IACUC#2015006).

### Vector Construction

The E1- and E3-deleted adenovirus serotype 5 (Ad5) was used as the vector for the delivery of target antigens. Replication-incompetent rAd5-G-H with open reading frames (ORFs) of RABV (SRV9 strain) G and CDV (CDV-11 strain) H genes, which were linked by the P2A gene, was constructed as previously described (Sullivan et al., 2000). rAd5-GFP, an adenovirus carrying green fluorescence protein (GFP) gene, served as a vector control. rAd5-G-H and rAd5-GFP were propagated in HEK-293 cells and purified by a round of CsCl gradient ultracentrifugation (Optima L-100 XP). Virus suspension was supplemented with 10% glycerol and stored at −80°C. Virus titers were determined in HEK-293 cells using the GFP-labeled method.

### Identification of Gene Expression

To confirm the expressions of RABV-G and CDV-H, HEK-293 cells were infected with purified rAd5-G-H at an MOI of 1. At 48 h (h) post infection (p.i.), the cells were washed with PBS and then fixed with 80% chilled acetone at −20°C. On the second day, the cells were washed three times with PBS and then incubated for 1 h with mouse anti-RABV G monoclonal antibody (1:300) or CDV polyclonal antibody (1:3000). Secondary antibodies of donkey anti-mouse IgG (1:1000) or donkey anti-goat IgG (1:1000) were used. The positive reaction signals were observed with an inverted fluorescence microscope (Olympus, IX71). The morphology and size of rAd5-G-H and rAd5-GFP were observed using transmission electron microscopy with negative staining.

### Mouse Vaccination and Challenge

Female BALB/c mice (6–8 weeks old) were divided into three groups (*n* = 32 in each group; Figure 2A). Two groups were injected intramuscular (i.m.) with 100 μl of supernatant containing 10^8^ GFU of rAd5-G-H or rAd5-GFP. The third group received 100 μl of DMEM by i.m. injection as a mock control. The mice were observed daily for signs of disease or death and for bodyweight measurement until 3 weeks after vaccination. The blood samples (*n*_1_ = 6 in each group) were randomly collected from the orbital vein at 1, 2, 4, and 8 weeks after immunization for determination of virus neutralization antibodies. The mice (*n*_2_ = 8 in each group) were challenged with 100-fold the 50% i.m. lethal dose (100 MLD_50_) of street RABV (HuNPB3 strain) by i.m. injection 4 weeks after vaccination. Mice were observed 21 days for clinical signs of rabies or death.

### Fox Vaccination and Challenge

Female foxes (8 weeks old) were divided into three groups (*n* = 6 in each group) (Figure 7A). Two groups were injected i.m. with 1 ml of supernatant containing 10^9^ GFU of rAd5-G-H or rAd5-GFP. The third group received 1 ml of DMEM by i.m. injection as mock control. The foxes were observed 3 weeks after vaccination for signs of disease or death. Blood samples were collected separately from the calf vein at 0, 2, 3, and 4 weeks after immunization for serological assessment. Four weeks after immunization, foxes in each group were challenged i.m. with 100-fold the 50% lethal dose (100 LD_50_) of wild-type CDVQL strain and observed 21 days for clinical signs of canine distemper.

### Neutralization Assays

The rabies VNAs in serum samples from the vaccinated mice and foxes were determined with a fluorescent antibody virus neutralization (FAVN) assay as previously described (Tian et al., 2015), and titers of rabies VNAs were expressed as IU/ml. The limited detection value of the FAVN assay was 0.02 IU/ml. Canine distemper VNAs were determined using the method of Appel and Robson (Appel and Robson, 1973); 50 μl of double serial diluted serum samples were pre-incubated with 50 μl of 100 TCID_50_ CDV-11 for 1 h and then added to 100 μl of 10^4^ Vero cells in each well in 96-well plates. The syncytium cytopathic effects (CPEs) were observed after 5 days. The titers of canine distemper VNAs were calculated by the methods of Reed and Muench (Reed and Muench, 1938).

### Specific Antibody Isotypes

Endpoint titers of antibodies of different subtypes (IgG1 and IgG2a) directed against purified and inactivated RABV and CDV antigens in serum samples from the rAd5-G-H group were determined with ELISA. Mouse serum samples from the mock group were considered to be free of RABV and CDV-specific IgG antibodies according to neutralization assays. In 96-well plates, the purified SRV9 or CDV-11 virions (10 μg/ml) were coated in PBS at 4°C overnight. After washing 3 times with PBS containing 0.05% Tween-20 (PBST), the plates were blocked with 1% BSA in PBS for 2 h at room temperature. After washing, 10-fold serially diluted sera from mice, starting from 1:10, were added to the plates, and they were incubated overnight at 4°C. Secondary antibodies of horseradish peroxidase (HRP)-labeled goat anti-mouse IgG1 or IgG2a (Southern Biotechnology, United States) were incubated at a dilution of 1:500 for 1 h at room temperature in the plates. After washing, substrate 2, 2′-azinobis-3-ethylbenzthiazoline-6-sulphonic acid (ABTS; Southern Biotechnology, Inc) was added and incubated for 15 min in plates, and the reaction was stopped with 1% SDS. Optical density (OD) at 405 nm was determined with a microplate reader (Mutiskan MK3, Thermo Fisher Scientific). Serum samples from mock mice were used to adjust the cutoff level. Endpoint titers were calculated as the reciprocals of the highest sera dilution that gave an OD at 405 nm 2.1 times higher than the cutoff value.

### Flow Cytometry

Flow cytometry was carried out to quantify the activations of immune cells in inguinal lymph nodes and in the peripheral blood. At 3, 6, and 9 days after vaccination, inguinal lymph nodes of the immunized mice (*n* = 6 in each group) were collected. A single lymphocyte suspension (10^6^ cells/ml) was prepared in 0.2% FBS 1640 (Gibco) and then stained with antibodies against CD19, CD40, CD11c, CD86, MHC I, and MHC II at 4°C for 30 min. At 7 and 14 days after vaccination, blood samples were collected from the immunized mice (*n* = 4 in each group). A single lymphocyte suspension (10^6^ cells/ml) was stained with antibodies against CD19 and CD40. Data were collected using the Beckman Coulter Cytoflex S flow cytometer (United States) and analyzed by CytExpert 2.0 software (Beckman Coulter).

### IFN-γ and IL-4 Enzyme-Linked Immunospot (ELISpot) Assays

Four weeks after vaccination, in 96-well ELISpot plates, lymphocytes from the spleen were isolated from the immunized mice (*n* = 4) and seeded in plates with 3 × 10^5^ cells per well. The lymphocytes were stimulated with the purified inactivated RABV and CDV at a concentration of 10 μg/ml. Secreted IFN-γ or IL-4 was quantitated using ELISpot assays (Dakewe Biotech Co., China) according to the manufacturer’s instructions. Spot-forming cells (SFCs) were counted with an ELISpot reader (AID ELISpot reader-iSpot, AID GmbH, GER).

### Statistical Analysis

Data are expressed as the mean ± standard deviation (SD). Statistical analyses were tested via Student’s *t*-test in SPSS 22.0 software (SPSS Inc., Chicago, IL, United States). Statistical significance was considered at ^∗^*P* < 0.05, ^∗∗^*P* < 0.01, and ^∗∗∗^*P* < 0.001.

## Results

### Construction and Characterization of rAd5-G-H

**FIGURE 1:**
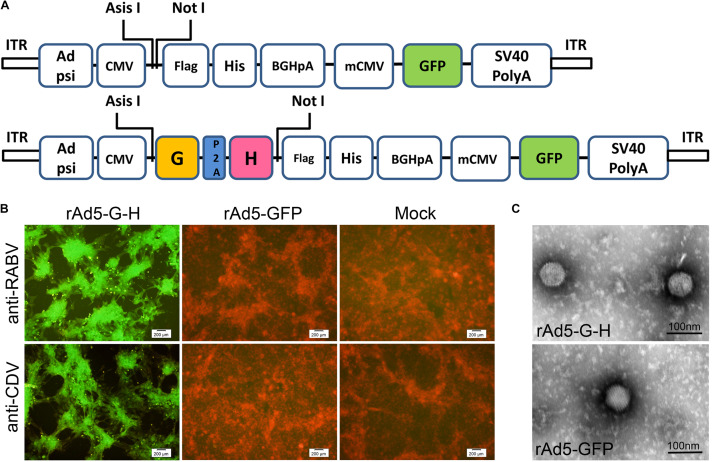
Generation and characterization of recombinant virus rAd5-G-H and rAd5-GFP. **(A)** Schematic representations of the rAd5-GFP and rAd5-G-H plasmids. **(B)** Immunofluorescence assay (IFA) analysis of RABV G protein and CDV H protein expression in HEK-293 cells infected with DMEM, rAd5-GFP, or rAd5-G-H (200× magnification). Monolayer HEK-293 cells were infected with purified rAd5-G-H or rAd5-GFP at an MOI of 1. Expressions of the G and H genes were examined 48 h p.i. with mouse anti-RABV G monoclonal antibody and goat anti-CDV polyclonal antibody, respectively. The secondary antibodies were an Alexa Fluor-488-conjugated donkey anti-rabbit IgG and donkey anti-goat IgG accordingly. **(C)** Morphologies of recombinant viruses rAd5-G-H and rAd5-GFP under transmission electron microscopy (30,000× magnification).

rAd5-G-H, a recombinant replication-incompetent adenovirus serotype 5 carrying the SRV9 G and CDV-11 H genes, was generated (Figure 1A). rAd5-GFP was an adenovirus carrying the GFP gene as a vector control. The inserted nucleotide components were confirmed by PCR and sequencing (data not shown). The expressions of the RABV G and CDV H genes in HEK-293 cells infected with rAd5-G-H, rAd5-GFP, or DMEM were detected by indirect immunofluorescence. Positive reaction signals against anti-RABV G monoclonal antibody and anti-CDV polyclonal serum were observed in rAd5-G-H-infected HEK-293 cells, while negative red signals were seen in HEK-293 cells infected with rAd5-GFP (Figure 1B).

The morphology of rAd5-G-H and rAd5-GFP under an electron microscope was fairly homogeneous and was icosahedral with an average diameter of 70 nm (Figure 1C). The surfaces of both recombinant virions appeared to be smooth. Therefore, both the RABV G and CDV H genes were co-expressed in rAd5-G-H-infected HEK-293 cells, and rAd5-G-H showed similar morphology, size, and surface appearance to the rAd5-GFP vector.

### Immunization With rAd5-G-H Protected Mice From Lethal Rabies Challenge

**FIGURE 2:**
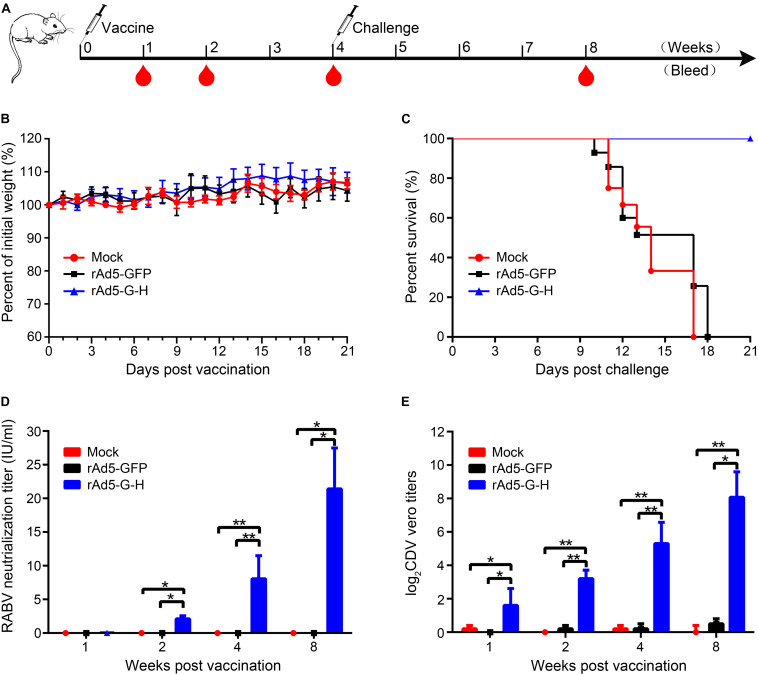
A single dose of rAd5-G-H elicited complete protection from lethal dose challenge with the RABV HuNPB3 strain in BALB/c mice. **(A)** Mouse study design. Female BALB/c mice (*n* = 32), 6–8 weeks old, were immunized i.m. with 10^8^ GFU rAd5-G-H or rAd5-GFP. Mice injected with DMEM served as mock controls. Some of the mice (*n*_1_ = 8) were challenged i.m. with 100 MLD_50_ street RABV HNPB3 strain 4 weeks after immunization. **(B)** Safety assessments. Mice were observed for 14 days for clinical signs and daily weights after immunization. **(C)** Survival curves after HNPB3 challenge in vaccinated mice. **(D,E)** At 1, 2, 4, and 8 weeks after immunization, blood samples (*n*_2_ = 6) were collected to detect specific virus-neutralizing antibodies to RABV **(D)** and CDV **(E)**. Statistical analyses were tested by Student’s t-test in SPSS 22.0 software. *P < 0.05, **P < 0.01, and ***P < 0.001.

Groups of mice were vaccinated i.m. with rAd5-G-H, rAd5-GFP, or DMEM. None of the mice showed abnormal behavior or neurological signs, and there were no significant changes in body weight among the three groups (Figure 2B). The rabies VNAs were detected only in serum samples from mice inoculated with rAd5-G-H, and the mean titers of rabies VNAs were 0.05, 2.12, 8.05, and 21.42 IU/ml at 1, 2, 4, and 8 weeks after immunity, respectively (Figure 2D). A VNA titer ≥0.5 IU/ml indicated adequate response to defend against a lethal RABV infection (Zhao et al., 2019). The mice vaccinated with rAd5-G-H demonstrated gradually increasing levels of VNAs against CDV from 1 to 8 weeks, and the average titers of VNAs were 1:5, 1:10, 1:59, and 1:473, respectively (Figure 2E). There were no detectable antibodies against either RABV or CDV in serum samples from mice inoculated with rAd5-GFP or DMEM (Figures 2D,E).

To evaluate the protection rates of rAd5-G-H against lethal RABV challenge, the mice were challenged with 100 MLD_50_ wild-type RABV HNPB_3_ strain 4 weeks after immunity. As shown in Figure 2C, all the mice vaccinated with rAd5-GFP and DMEM died within 21 days, as expected; however, those immunized with rAd5-G-H all survived without any clinical signs of rabies. Exclusively, RABV N genes were further confirmed by RT-PCR and sequencing in the brains of all the mice who died (Supplementary Figure S1). Thus, the rAd5-G-H elicited robust and persistent humoral immunity against both RABV and CDV in mice and provided 100% protection against lethal RABV infections.

### Specific Antibody Isotypes

**FIGURE 3:**
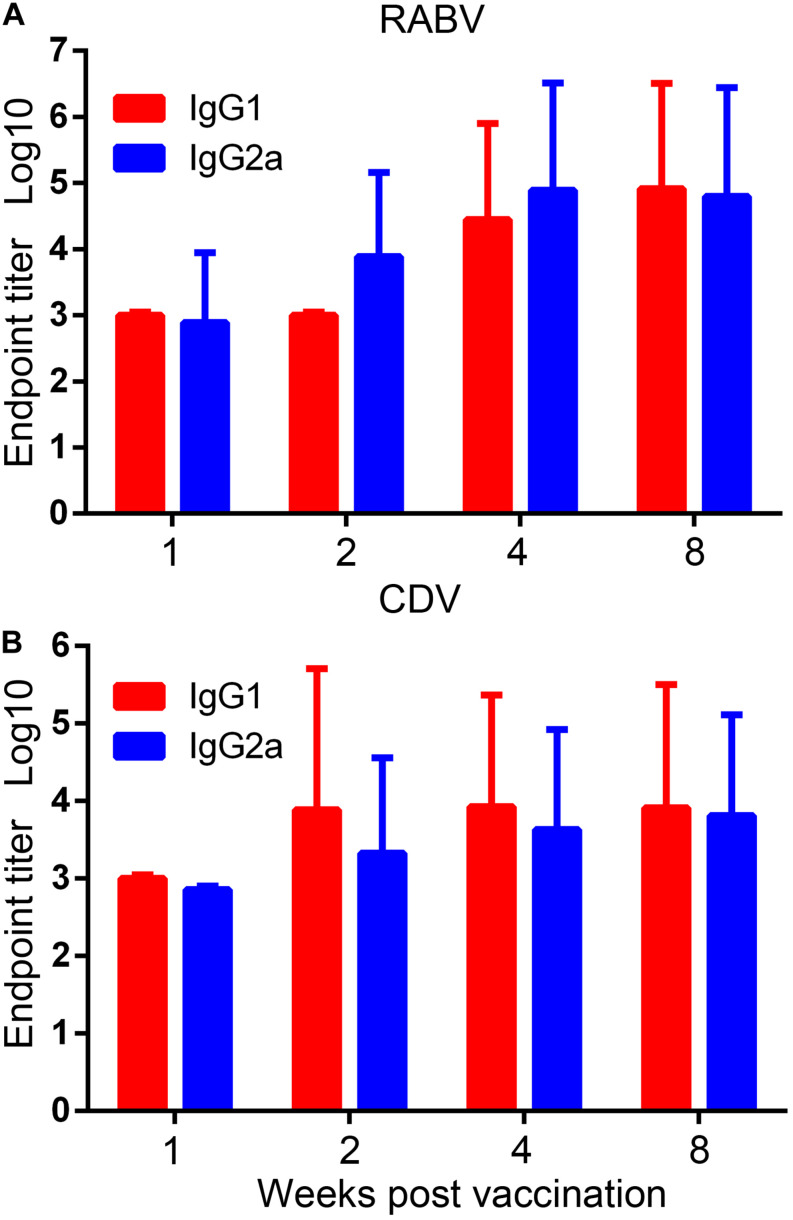
Virus-specific IgG1 and IgG2a subtype responses. Endpoint titers of IgG1 and IgG2a subtypes in serum samples of the rAd5-G-H group were detected with purified inactivated RABV and CDV by ELISA. Endpoint titers were calculated as the reciprocals of the highest serum dilution that gave an OD at 405 nm 2.1 times higher than the cutoff value. **(A)** RABV-specific and **(B)** CDV-specific IgG1 and IgG2a subtype responses.

Specific IgG2a and IgG1 antibodies, the markers of Th1 and Th2 responses, respectively (Mountford et al., 1994), in serum samples of the rAd5-G-H-vaccinated mice against RABV or CDV were examined by ELISA assays. Titers of IgG1 and IgG2a against RABV showed robust elevations after 4 weeks and reached higher levels of 1:82,000 and 1:64,000 at 8 weeks, respectively (Figure 3A). Against CDV, a rapid and higher level of IgG1 was elicited than that of IgG2a 2 weeks after immunity; however, the titers of both isotypes were maintained at approximately 1:8200 and 1:6512 at 8 weeks, respectively (Figure 3B). Notably, the antibody responses of IgG1 and IgG2a against RABV were much higher than those against CDV at the same time points. The ratio of IgG1/IgG2a against both RABV and CDV almost equaled 1, which suggested that rAd5-G-H induced both Th1 and Th2 immune responses in mice.

### Recruitment and/or Activation of Dendritic Cells and B Cells by rAd5-G-H

**FIGURE 4:**
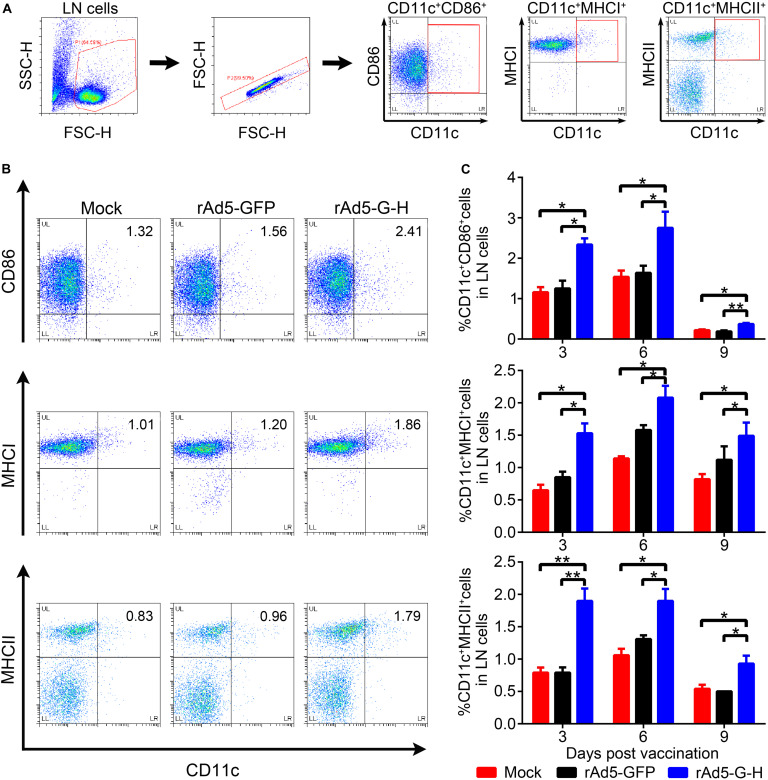
Recruitment and/or activation of DCs in mice vaccinated with rAd5-G-H. BALB/c mice (*n* = 6) were immunized i.m. with 10^8^ GFU rAd5-G-H, rAd5-GFP or DMEM. The inguinal lymph nodes (LNs) were collected at 3, 6, and 9 days after vaccination. Single-cell suspensions were prepared and stained with antibodies of DC and then analyzed by flow cytometry. **(A,B)** The gating strategies for analyzing the DCs **(A)** and representative flow cytometric plots for measuring recruited and/or activated DCs **(B)** are shown. **(C)** Analyses for activated DCs (CD11c^+^ CD86^+^, CD11c^+^ MHCI^+^, CD11c^+^ MHCII^+^) from the LNs at 3, 6, and 9 days after vaccination are presented. Statistical analyses were tested by Student’s *t*-test in SPSS 22.0 software. *P < 0.05, **P < 0.01, and ***P < 0.001.

**FIGURE 5:**
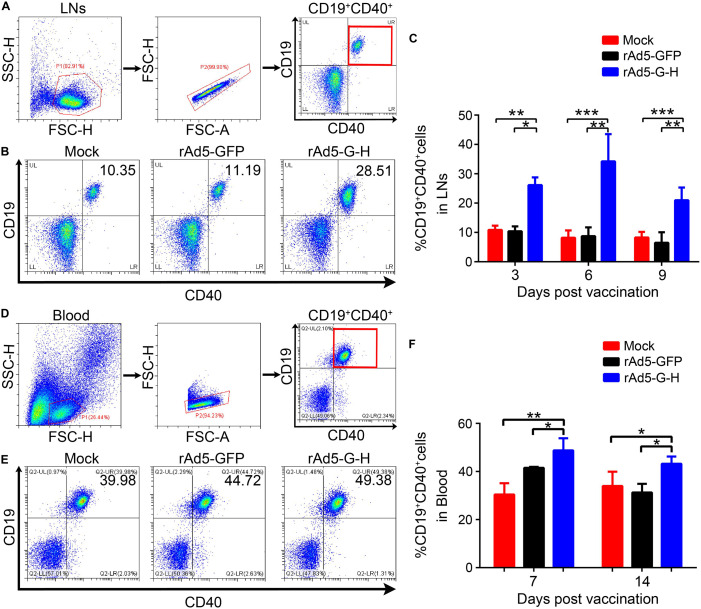
Recruitments and/or activations of B cells from LNs and peripheral blood in mice vaccinated with rAd5-G-H. BALB/c mice (*n* = 10 in each group) were immunized i.m. with 10^8^ GFU rAd5-G-H, rAd5-GFP, or DMEM. Single-cell suspensions of LNs (*n* = 6) were collected and prepared at 3, 6, and 9 dpi, and those from peripheral blood (*n* = 4) were collected at 7 and 14 dpi. The cells were stained with antibodies to CD19 and CD40 and then analyzed by flow cytometry. **(A,D)** The gating strategies for analyzing the B cells and **(B,E)** representative flow cytometric plots for measuring recruited and/or activated B cells (CD19^+^ CD40^+^) are shown. Analyses of activated B cells from the LNs **(C)** at 3, 6, and 9 dpi and from the peripheral blood **(F)** at 7 and 14 dpi are presented. Statistical analyses were tested by Student’s *t*-test in SPSS 22.0 software. *P < 0.05, **P < 0.01, and ***P < 0.001.

To investigate whether rAd5-G-H recruited dendritic cells (DCs) and activated B cells *in vivo*, the percentages of DCs (CD11c^+^ CD86^+^, CD11c^+^ MHC I^+^, CD11c^+^ MHC II^+^) and B cells (CD19^+^ CD40^+^) from the inguinal lymph nodes of mice (*n* = 6) were analyzed by flow cytometry at 3, 6, and 9 days post immunization (dpi), and the percentages of B cells (CD19^+^ CD40^+^) in the peripheral blood of mice (*n* = 4) were analyzed at 7 and 14 dpi. The gating strategies for analyzing the DCs and representative flow cytometric plots for measuring recruited and/or activated DCs are shown in Figures 4A,B, respectively. As shown in Figure 4C, in comparison with the rAd5-GFP and DMEM groups, more DCs (CD11c^+^ CD86^+^, CD11c^+^ MHC I^+^, CD11c^+^ MHC II^+^) were significantly recruited in the inguinal lymph nodes of the mice immunized with rAd5-G-H at corresponding time points (3, 6, and 9 dpi). Figure 5 shows the gating strategies for analyzing the B cells (Figures 5A,D) and representative flow cytometric plots for measuring recruited and/or activated B cells (Figures 5B,E). As shown in Figure 5C, significantly more B cells (CD19^+^ CD40^+^) were detected in LNs from mice immunized with rAd5-G-H than in those from mice immunized with rAd5-GFP and DMEM at 3, 6, and 9 dpi. As shown in Figure 5F, significantly more B cells (CD19^+^ CD40^+^) were detected in the peripheral blood of mice immunized with rAd5-G-H compared to those in mice immunized with rAd5-GFP and DMEM at 7 and 14 dpi. Thus, rAd5-G-H recruited and/or activated more DCs to stimulate MHC I and MHC II expression and induced persistent B-cell activation to develop robust and lasting immune responses.

### Specific T Cell-Mediated Immune (CMI) Responses in Mice

**FIGURE 6:**
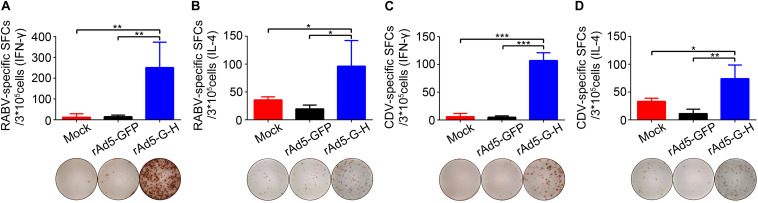
RABV- and CDV-specific T cell-mediated immune (CMI) responses in mice. BALB/c mice (*n* = 4) vaccinated i.m. with rAd5-G-H, rAd5-GFP, or DMEM were sacrificed 4 weeks after vaccination. Splenocytes were stimulated with the purified inactivated RABV and CDV. The levels of secreted IFN-γ and IL-4 were detected using ELISpot analysis. **(A)** RABV-specific IFN-γ SFCs. **(B)** RABV-specific IL-4 SFCs. **(C)** CDV-specific IFN-γ SFCs. **(D)** CDV-specific IL-4 SFCs. Representative images for IFN-γ or IL-4 in each group are shown below the graphs. Statistical analyses were tested by Student’s *t*-test in SPSS 22.0 software. *P < 0.05, **P < 0.01, and ***P < 0.001.

The IFN-γ and IL-4 ELISpot assays showed that rAd5-G-H induced significantly higher levels of RABV-specific IFN-γ and IL-4 SFCs (Figures 6A,B) and CDV-specific IFN-γ and IL-4 SFCs (Figures 6C,D) 4 weeks after immunity in mice in comparison with the rAd5-GFP and mock groups. Notably, there were more IFN-γ-specific SFCs than IL-4-specific SFCs against both RABV and CDV. These findings indicated that rAd5-G-H elicited Th1 preferred immune responses in mice, which contributed to the 100% protection against both RABV and CDV infections.

### Immunization With rAd5-G-H Protected Foxes From Lethal CDV Challenge

**FIGURE 7:**
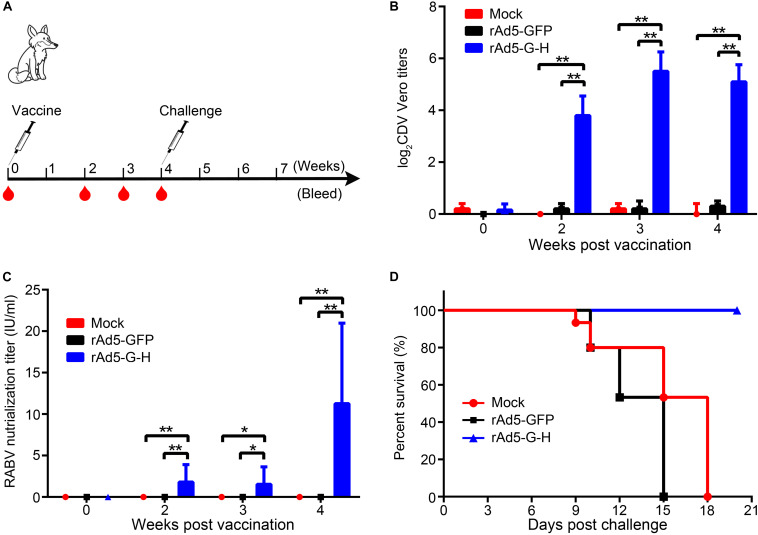
A single dose of rAd5-G-H elicited complete protection against challenge with a lethal dose of CDVQL strain in foxes. **(A)** Fox study design. Eight-week-old female foxes (*n* = 6) were immunized i.m. with 10^9^ GFU rAd5-G-H or rAd5-GFP. Foxes injected with DMEM served as mock controls. Foxes were challenged i.m. with 100 LD_50_ of wild-type CDVQL strain 4 weeks after immunization. **(B,C)** At 0, 2, 3, and 4 weeks after immunization, blood samples were collected to detect VNAs against RABV **(B)** and CDV **(C)**. **(D)** Survival curves of foxes challenged with CDVQL. Statistical analyses were tested by Student’s *t*-test in SPSS 22.0 software. *P < 0.05, **P < 0.01, and ***P < 0.001.

To evaluate the immune effects of rAd5-G-H in carnivores, the foxes were vaccinated with rAd5-G-H and challenged with wild-type CDVQL strain. As shown in Figure 7B, the mean titers of canine distemper VNAs in rAd5-G-H-immunized mice were 1:16 at 2 weeks and were maintained at approximately 1:60 after 3 weeks. Meanwhile, rabies VNAs were considerably induced, with titers up to 11.26 IU/ml at 4 weeks in rAd5-G-H-immunized foxes (Figure 7C). Neither CDV- nor RABV-neutralizing antibodies were detected in foxes inoculated with rAd5-GFP and DMEM.

Three groups of foxes were challenged with 100 LD_50_ of the pathogenic CDV QL strain 4 weeks after vaccination. As shown in Figure 7D, all foxes (6 of 6) immunized with rAd5-G-H survived, while the foxes inoculated with rAd5-GFP (6 of 6) and DMEM (6 of 6) succumbed to the fatal CDV infection. Exclusively, CDV N genes in the lungs of all foxes who died were positive according to RT-PCR (Supplementary Figure S2). These results demonstrated that rAd5-G-H was immunogenic in foxes and protected them from lethal CDV challenge.

## Discussion

Rabies vaccines have been inactivated to make them completely safe (da Fontoura Budaszewski et al., 2017). However, many studies have shown that 30% of dogs failed to reach a VNA titer of 0.5 IU/ml with one dose of commercial inactivated rabies vaccine (Zhang W. et al., 2019). Modified live vaccines (MLVs) and canarypox-vectored recombinant vaccines against CDV have been applied commercially to protect carnivores for many years (Georoff, 2019). However, MLVs might cause severe diseases and death in highly susceptible species (Barrett, 1999). There have been many cases of canine distemper outbreaks in vaccinated animals (Hartley, 1974; Ek-Kommonen et al., 1997). The immunity failure was mainly caused by inadequate immune responses to CDV MLVs in the presence of maternal antibodies in puppies (Stephensen et al., 1997).

It was necessary to develop a bivalent vaccine against rabies and canine distemper. A recombinant CDV rCDV-RABVG, expressing glycoprotein of the LEP-Flury strain, induced specific VNAs against RABV G and CDV in vaccinated mice (Li et al., 2015); however, there was no protective evaluation in susceptible animals. Another rCDV-RABVG generated by reverse genetics induced VNAs to RABV and CDV in dogs (Wang et al., 2012). However, it required multiple immunizations to achieve high levels of antibody responses. A recombinant RABV rLBNSE-CDVH, expressing CDV H protein, protected dogs from a lethal wild-type CDV challenge (Wang et al., 2014). These bivalent vaccines were all live-attenuated vaccines with risks in highly susceptibility species. Based on an avirulent RABV vector, BNSP-333, the inactivated recombinant RABVs expressing CDV H or CDV F protected against lethal CDV challenge with mixed immunity in ferrets immunized twice (da Fontoura Budaszewski et al., 2017).

Adenoviruses serve as vectors of gene delivery and expression, and human adenovirus serotype 5 is one of the most widely used recombinant vectors, with several advantages (Tatsis and Ertl, 2004; Wold and Toth, 2013). Deletion of E1 resulted in a replication-deficient recombinant adenovirus unless in E1 complementary cells, which increased its safety (Wold and Toth, 2013). The rAd5 vector is easy to purify and has a high growth titer in HEK 293 cells under good manufacturing practice (GMP) conditions, which reduces the costs of vaccines (Liu et al., 2009). Importantly, adenoviruses had species-specificity and different serotypes, and our bivalent vaccine targeted dogs and other carnivores, which had low pre-existing neutralizing antibodies to AdHu5 (Tatsis and Ertl, 2004).

In the present study, based on an E1- and E3-deficient rAd5 vector, rAd5-G-H co-expressing the RABV G and CDV H genes was generated as a potential bivalent vaccine against RABV and CDV. The porcine teschovirus-1 2A (P2A) sequence connected and mediated “cleavage” between the RABV G and CDV H genes for simultaneous expression. Compared with the internal ribosome entry site (IRES), P2A is smaller in size and has greater cleavage efficiency. Researchers have proven that rAd5 vectors induce faster and higher levels of protective neutralizing antibodies against the transgene product than DNA vaccines after a single dose (Sullivan et al., 2003; Tatsis and Ertl, 2004). However, in the current study, the titers of rabies VNAs were 2.12, 8.05, and 21.42 IU/ml at 2, 4, and 8 weeks, respectively, after a single-dose immunization of rAd5-G-H in mice, which significantly exceeded the minimum protection level of the WHO (0.5 IU/ml) (World Health Organization, 2013). In foxes, titers of rabies VNAs reached 11.26 IU/ml at 8 weeks with single-dose immunity, which is strong evidence of effective immune protection. Titers of canine distemper VNAs reached 1:453 at 8 weeks in mice and 1:38 at 4 weeks in foxes after vaccination with rAd5-G-H, which indicated strong humoral immune responses against CDV.

Numerous studies have evidenced that replication-incompetent adenovirus vectors elicited both antibody and T-cell immune responses (Gao et al., 2006; Ledgerwood et al., 2010; Smaill et al., 2013). An E1- and E3-deleted Ad5-based influenza A virus vaccine induced both HA-specific antibodies and cellular immunity in BALB/c mice (Gao et al., 2006). A replication-defective virus, rAd5-EbolaG, induced durable neutralizing antibodies and CMI responses in non-human primates (Ledgerwood et al., 2010). A human Ad5-based tuberculosis vaccine induced robust T-cell responses in humans (Smaill et al., 2013). In this study, the percentages of CD11c^+^ CD86^+^, CD11c^+^ MHCI^+^, CD11c^+^ MHCII^+^, and CD19^+^ CD40^+^ from LNs and peripheral blood were notably increased in mice vaccinated with rAd5-G-H as time went on compared with those in the rAd5-GFP and mock mouse groups. rAd5-G-H recruited more DCs and/or activated more persistent B cells, the major antigen-presenting cells, which played critical roles in enhancing humoral immune responses. The isotypes of IgG1 and IgG2a maintained high levels against RABV and CDV in rAd5-G-H-immunized mice. The ratio of IgG1/IgG2a almost equaled 1, suggesting that the rAd5-G-H induced both Th1 and Th2 immune responses in mice. IFN-γ is a Th1-type cytokine involved in the antiviral action of cellular immune responses, while IL-4 is mainly produced by Th2 cells and has been associated with humoral immune responses (Zhang S. et al., 2019). Notably, the rAd5-G-H induced more IFNγ^+^ SFCs than IL-4^+^ SFCs 4 weeks after immunity in mice, indicating a Th1 preferred cellular-mediated immune response, which contributed to a 100% protection against RABV challenge in mice and CDV challenge in foxes. These results indicated that rAd5-G-H was beneficial to the antigen presentation of RABV G and CDV H protein and elicited antiviral humoral and cellular immune responses.

In conclusion, a recombinant replication-defective virus, rAd5-G-H, co-expressing the RABV G and the CDV H proteins, was generated. A single dose of rAd5-G-H induced robust and rapid humoral and cell-mediated immunities against RABV and CDV, with 100% protection against a lethal RABV challenge in mice. In addition, a single dose of rAd5-G-H elicited high levels of VNAs against CDV and protected foxes from fatal CDV challenge. rAd5-G-H has great potential as a bivalent vaccine for protection against RABV and CDV in carnivores.

## Data Availability Statement

All datasets generated for this study are included in the article/Supplementary Material.

## Ethics Statement

The animal study was reviewed and approved by Institutional Animal Care and Use Committee (IACUC#2015006).

## Author Contributions

LY and ZZ performed the experiments. XX and WZ edited the manuscript. TX, LL, and LT analyzed the data. XW prepared the figures. HH helped explain the data. XZ designed the study. All authors contributed to the manuscript and approved the submitted version.

## Conflict of Interest

The authors declare that the research was conducted in the absence of any commercial or financial relationships that could be construed as a potential conflict of interest.

## References

[B1] Appel M. Robson D. S. (1973). A microneutralization test for canine distemper virus. *Am. J. Vet. Res.* 34 1459–1463. 4201293

[B2] Astray R. M. Jorge S. A. Pereira C. A. (2017). Rabies vaccine development by expression of recombinant viral glycoprotein. *Arch. Virol.* 162 323–332. 10.1007/s00705-016-3128-9 27796547

[B3] Barrett T. (1999). Morbillivirus infections, with special emphasis on morbilliviruses of carnivores. *Vet. Microbiol.* 69 3–13. 10.1016/s0378-1135(99)00080-2 10515262

[B4] Bhatt M. Rajak K. K. Chakravarti S. Yadav A. K. Kumar A. Gupta V. (2019). Phylogenetic analysis of haemagglutinin gene deciphering a new genetically distinct lineage of canine distemper virus circulating among domestic dogs in India. *Transbound. Emerg. Dis.* 66 1252–1267. 10.1111/tbed.13142 30725534

[B5] Churchyard G. J. Morgan C. Adams E. Hural J. Graham B. S. Moodie Z. (2011). A Phase IIA Randomized Clinical Trial of a Multiclade HIV-1 DNA Prime Followed by a Multiclade rAd5 HIV-1 Vaccine Boost in Healthy Adults (HVTN204). *PLoS One* 6:e21225. 10.1371/journal.pone.0021225 21857901 PMC3152265

[B6] da Fontoura Budaszewski R. Hudacek A. Sawatsky B. Krämer B. Yin X. Schnell M. J. (2017). Inactivated Recombinant Rabies Viruses Displaying Canine Distemper Virus Glycoproteins Induce Protective Immunity against Both Pathogens. *J. Virol.* 91 e2077–e2016. 10.1128/JVI.02077-16 28148801 PMC5375678

[B7] Deem S. L. Spelman L. H. Yates R. A. Montali R. J. (2000). Canine distemper in terrestrial carnivores: a review. *J. Zoo Wildl. Med.* 31 441–451. 10.1638/1042-7260(2000)031[0441:cditca]2.0.co;2 11428391

[B8] Ek-Kommonen C. Sihvonen L. Pekkanen K. Rikula U. Nuotio L. (1997). Outbreak off canine distemper in vaccinated dogs in Finland. *Vet. Rec.* 141 380–383. 10.1136/vr.141.15.380 9364705

[B9] El-Sayed A. (2018). Advances in rabies prophylaxis and treatment with emphasis on immunoresponse mechanisms. *Int. J. Vet. Sci. Med.* 6 8–15. 10.1016/j.ijvsm.2018.05.001 30255072 PMC6149183

[B10] Feng N. Yu Y. Wang T. Wilker P. Wang J. Li Y. (2016). Fatal canine distemper virus infection of giant pandas in China. *Sci. Rep.* 6:27518. 10.1038/srep27518 27310722 PMC4910525

[B11] Gao W. Soloff A. C. Lu X. Montecalvo A. Nguyen D. C. Matsuoka Y. (2006). Protection of Mice and Poultry from Lethal H5N1 Avian Influenza Virus through Adenovirus-Based Immunization. *J. Virol.* 80 1959–1964. 10.1128/jvi.80.4.1959-1964.2006 16439551 PMC1367171

[B12] Georoff T. A. (2019). “Canine distemper vaccination in nondomestic carnivores,” in *Fowler’s Zoo and Wild Animal Medicine Current Therapy*, ed. Miller E. R. (Amsterdam: Elsevier), 555–563. 10.1016/b978-0-323-55228-8.00079-5

[B13] Guo X. Deng Y. Chen H. Lan J. Wang W. Zou X. (2015). Systemic and mucosal immunity in mice elicited by a single immunization with human adenovirus type 5 or 41 vector-based vaccines carrying the spike protein of Middle East respiratory syndrome coronavirus. *Immunology* 145 476–484. 10.1111/imm.12462 25762305 PMC4515128

[B14] Hartley W. J. (1974). A post-vaccinal inclusion body encephalitis in dogs. *Vet. Pathol.* 11 301–312. 10.1177/030098587401100403 4156617

[B15] Jackson A. C. (2013). Current and future approaches to the therapy of human rabies. *Antiviral Res.* 99 61–67. 10.1016/j.antiviral.2013.01.003 23369672

[B16] Khanam S. Pilankatta R. Khanna N. Swaminathan S. (2009). An adenovirus type 5 (AdV5) vector encoding an envelope domain III-based tetravalent antigen elicits immune responses against all four dengue viruses in the presence of prior AdV5 immunity. *Vaccine* 27 6011–6021. 10.1016/j.vaccine.2009.07.073 19665609

[B17] Ledgerwood J. E. Costner P. Desai N. Holman L. Enama M. E. Yamshchikov G. (2010). A replication defective recombinant Ad5 vaccine expressing Ebola virus GP is safe and immunogenic in healthy adults. *Vaccine* 29 304–313. 10.1016/j.vaccine.2010.10.037 21034824

[B18] Li S. Yi L. Cao Z. Cheng Y. Tong M. Wang J. (2018). Identification of linear B-cell epitopes on the phosphoprotein of canine distemper virus using four monoclonal antibodies. *Virus Res.* 257 52–56. 10.1016/j.virusres.2018.08.021 30213628

[B19] Li Z. Wang J. Yuan D. Wang S. Sun J. Yi B. (2015). A recombinant canine distemper virus expressing a modified rabies virus glycoprotein induces immune responses in mice. *Virus Genes* 50 434–441. 10.1007/s11262-015-1169-x 25764477

[B20] Liu H. Liu X. M. Li S. C. Wu B. C. Ye L. L. Wang Q. W. (2009). A high-yield and scaleable adenovirus vector production process based on high density perfusion culture of HEK 293 cells as suspended aggregates. *J. Biosci. Bioeng.* 107 524–529. 10.1016/j.jbiosc.2009.01.004 19393552

[B21] Martinez-Gutierrez M. Ruiz-Saenz J. (2016). Diversity of susceptible hosts in canine distemper virus infection: a systematic review and data synthesis. *BMC Vet. Res.* 12:78. 10.1186/s12917-016-0702-z 27170307 PMC4865023

[B22] Mountford A. P. Fisher A. Wilson R. A. (1994). The profile of IgG1 and IgG2a antibody responses in mice exposed to *Schistosoma mansoni*. *Parasite Immunol.* 16 521–527. 10.1111/j.1365-3024.1994.tb00306.x 7870462

[B23] Noyce R. S. Delpeut S. Richardson C. D. (2013). Dog nectin-4 is an epithelial cell receptor for canine distemper virus that facilitates virus entry and syncytia formation. *Virology* 436 210–220. 10.1016/j.virol.2012.11.011 23260107

[B24] Reed L. J. Muench H. (1938). A simple method of eatimating fifty per cent endpoints. *Am. J. Epidemiol.* 27 493–497. 10.1093/oxfordjournals.aje.a118408

[B25] Rendon-Marin S. da Fontoura Budaszewski R. Canal C. W. Ruiz-Saenz J. (2019). Tropism and molecular pathogenesis of canine distemper virus. *Virol. J.* 16:30. 10.1186/s12985-019-1136-6 30845967 PMC6407191

[B26] Romanutti C. Gallo Calderon M. Keller L. Mattion N. La Torre J. (2016). RT-PCR and sequence analysis of the full-length fusion protein of Canine Distemper Virus from domestic dogs. *J. Virol. Methods* 228 79–83. 10.1016/j.jviromet.2015.11.011 26611227

[B27] Scott T. P. Nel L. H. (2016). Subversion of the immune response by rabies virus. *Viruses* 8:E231. 10.3390/v8080231 27548204 PMC4997593

[B28] Smaill F. Jeyanathan M. Smieja M. Medina M. F. Thanthrige-Don N. Zganiacz A. (2013). A human type 5 adenovirus–based tuberculosis vaccine induces robust T cell responses in humans despite preexisting anti-adenovirus immunity. *Sci. Transl. Med.* 5:205ra134. 10.1126/scitranslmed.3006843 24089406

[B29] Stephensen C. B. Welter J. Thaker S. R. Taylor J. Tartaglia J. Paoletti E. (1997). Canine distemper virus (CDV) infection of ferrets as a model for testing Morbillivirus vaccine strategies: NYVAC- and ALVAC-based CDV recombinants protect against symptomatic infection. *J. Virol.* 71 1506–1513. 10.1128/jvi.71.2.1506-1513.1997 8995676 PMC191207

[B30] Sullivan N. J. Geisbert T. W. Geisbert J. B. Xu L. Yang Z. Y. Roederer M. (2003). Accelerated vaccination for Ebola virus haemorrhagic fever in non-human primates. *Nature* 424 681–684. 10.1038/nature01876 12904795 PMC7095492

[B31] Sullivan N. J. Sanchez A. Rollin P. E. Yang Z. Y. Nabel G. J. (2000). Development of a preventive vaccine for Ebola virus infection in primates. *Nature* 408 605–609. 10.1038/35046108 11117750

[B32] Tatsis N. Ertl H. C. (2004). Adenoviruses as vaccine vectors. *Mol. Ther.* 10 616–629. 10.1016/j.ymthe.2004.07.013 15451446 PMC7106330

[B33] Tatsuo H. Ono N. Yanagi Y. (2001). Morbilliviruses use signaling lymphocyte activation molecules (CD150) as cellular receptors. *J. Virol.* 75 5842–5850. 10.1128/JVI.75.13.5842-5850.2001 11390585 PMC114299

[B34] Tian D. Luo Z. Zhou M. Li M. Yu L. Wang C. (2015). Critical Role of K1685 and K1829 in the large protein of rabies virus in viral pathogenicity and immune evasion. *J. Virol.* 90 232–244. 10.1128/JVI.02050-15 26468538 PMC4702542

[B35] von Messling V. Milosevic D. Cattaneo R. (2004). Tropism illuminated: lymphocyte-based pathways blazed by lethal morbillivirus through the host immune system. *Proc. Natl. Acad. Sci. U.S.A.* 101 14216–14221. 10.1073/pnas.0403597101 15377791 PMC521139

[B36] Vos A. Neubert A. Pommerening E. Müller T. Döhner L. Neubert L. (2001). Immunogenicity of an E1-deleted recombinant human adenovirus against rabies by different routes of administration. *J. Gen. Virol.* 82 2191–2197. 10.1099/0022-1317-82-9-2191 11514729

[B37] Wang F. X. Zhang S. Q. Zhu H. W. Yang Y. Sun N. Tan B. (2014). Recombinant rabies virus expressing the H protein of canine distemper virus protects dogs from the lethal distemper challenge. *Vet. Microbiol.* 174 362–371. 10.1016/j.vetmic.2014.10.023 25465178

[B38] Wang X. Feng N. Ge J. Shuai L. Peng L. Gao Y. (2012). Recombinant canine distemper virus serves as bivalent live vaccine against rabies and canine distemper. *Vaccine* 30 5067–5072. 10.1016/j.vaccine.2012.06.001 22698451

[B39] Wirblich C. Schnell M. J. (2011). Rabies virus (RV) glycoprotein expression levels are not critical for pathogenicity of RV. *J. Virol.* 85 697–704. 10.1128/JVI.01309-10 21068252 PMC3020019

[B40] Wold W. S. M. Toth K. (2013). Adenovirus vectors for gene therapy, vaccination and cancer gene therapy. *Curr. Gene Ther.* 13 421–433. 10.2174/1566523213666131125095046 24279313 PMC4507798

[B41] World Health Organization (2013). WHO Expert Consultation on Rabies. Second report. *World Health Organ Tech. Rep. Ser.* 982 1–139. 24069724

[B42] World Health Organization (2019). *Rabies. Epidemiology and burden of disease [Online].* Geneva: WHO.

[B43] Xue X. Zhu Y. Yan L. Wong G. Sun P. Zheng X. (2019). Antiviral efficacy of favipiravir against canine distemper virus infection in vitro. *BMC Vet. Res.* 15:316. 10.1186/s12917-019-2057-8 31477101 PMC6720089

[B44] Zhang S. Hao M. Feng N. Jin H. Yan F. Chi H. (2019). Genetically modified rabies virus vector-based rift valley fever virus vaccine is safe and induces efficacious immune responses in mice. *Viruses* 11:919. 10.3390/v11100919 31597372 PMC6832564

[B45] Zhang W. Cheng N. Wang Y. Zheng X. Zhao Y. Wang H. (2019). Adjuvant activity of PCP-II, a polysaccharide from Poria cocos, on a whole killed rabies vaccine. *Virus Res.* 270:197638. 10.1016/j.virusres.2019.06.001 31173772

[B46] Zhao R. Yu P. Shan Y. Thirumeni N. Li M. Lv Y. (2019). Rabies virus glycoprotein serology ELISA for measurement of neutralizing antibodies in sera of vaccinated human subjects. *Vaccine* 37 6060–6067. 10.1016/j.vaccine.2019.08.043 31471146

[B47] Zhu F. Wurie A. H. Hou L. Liang Q. Li Y. Russell J. B. W. (2017). Safety and immunogenicity of a recombinant adenovirus type-5 vector-based Ebola vaccine in healthy adults in Sierra Leone: a single-centre, randomised, double-blind, placebo-controlled, phase 2 trial. *Lancet* 389 621–628. 10.1016/s0140-6736(16)32617-4 28017399

