# Supplementary material for: A Bivalent Human Adenovirus Type 5 Vaccine Expressing the Rabies Virus Glycoprotein and Canine Distemper Virus Hemagglutinin Protein Confers Protective Immunity in Mice and Foxes
Source: Front Microbiol. 2020 Jun 16;11:1070. doi: 10.3389/fmicb.2020.01070 (PMC7309451; doi:10.3389/fmicb.2020.01070)
Supplement: Supplementary file 1 [file Data_Sheet_1.pdf]

Supplementary Figures

The RT-PCR results for RABV N genes from the brains of all deceased mice are shown in Figure S1.

RT-PCR was conducted on the lungs of the deceased foxes to identify CDV N gene fragments, and the results are shown in Figure S2.

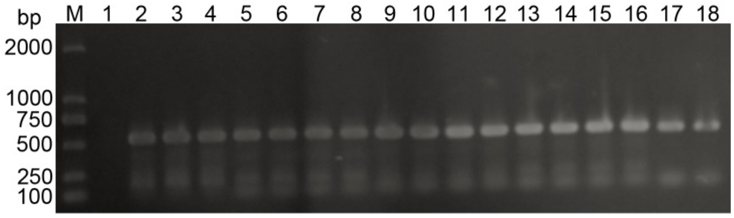

Figure S1. RT-PCR results for RABV N genes in the brains of all deceased mice.

Lane 1 is negative control; Lane 18 is the positive amplification from RABV culture; Lanes 2–9 are the RT-PCR results from brains of mice inoculated with the rAd-GFP strain; Lanes 10–17 are the RT-PCR results for the brains of mice incubated with DMEM.

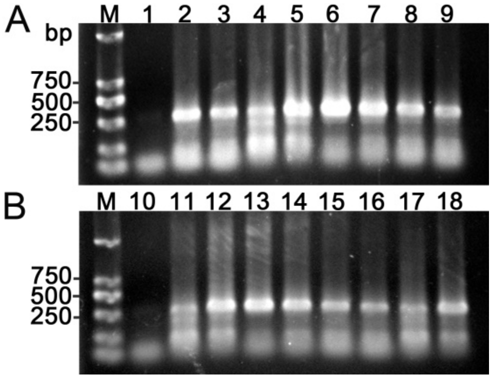

Figure S2. RT-PCR results for the lungs of deceased foxes.

Lane 1 and Lane 10 are negative control; Lanes 8–9 and 17–18 are positive amplifications from the CDV culture; Lanes 2–7 are RT-PCR results for the lungs of foxes inoculated with the rAd-GFP strain; Lanes 11–16 are RT-PCR results for the lungs of foxes inoculated with DMEM.
